# Historical Contingency Causes Divergence in Adaptive Expression of the *lac* Operon

**DOI:** 10.1093/molbev/msab077

**Published:** 2021-03-21

**Authors:** Kedar Karkare, Huei-Yi Lai, Ricardo B.R. Azevedo, Tim F. Cooper

**Affiliations:** 1 Department of Biology and Biochemistry, University of Houston, Houston, TX, USA; 2 School of Natural and Computational Sciences, Massey University, Auckland, New Zealand

**Keywords:** adaptation, epistasis, gene expression, *lac* operon, experimental evolution

## Abstract

Populations of *Escherichia coli* selected in constant and fluctuating environments containing lactose often adapt by substituting mutations in the *lacI* repressor that cause constitutive expression of the *lac* operon. These mutations occur at a high rate and provide a significant benefit. Despite this, eight of 24 populations evolved for 8,000 generations in environments containing lactose contained no detectable repressor mutations. We report here on the basis of this observation. We find that, given relevant mutation rates, repressor mutations are expected to have fixed in all evolved populations if they had maintained the same fitness effect they confer when introduced to the ancestor. In fact, reconstruction experiments demonstrate that repressor mutations have become neutral or deleterious in those populations in which they were not detectable. Populations not fixing repressor mutations nevertheless reached the same fitness as those that did fix them, indicating that they followed an alternative evolutionary path that made redundant the potential benefit of the repressor mutation, but involved unique mutations of equivalent benefit. We identify a mutation occurring in the promoter region of the *uspB* gene as a candidate for influencing the selective choice between these paths. Our results detail an example of historical contingency leading to divergent evolutionary outcomes.

## Introduction

“I am [speaking] of the central principle of all history—contingency. A historical explanation [rests] on an unpredictable sequence of antecedent states, where any major change in any step of the sequence would have altered the final result. This final result is therefore dependent, or contingent, upon everything that came before—the unerasable and determining signature of history.”

        —Stephen J. Gould (1989) *Wonderful Life*, p. 283.

“Life is just one damned thing after another!”

        —Lilian Bell (1909) *The Concentrations of Bee*, p. 241. The expression was used widely at that time.

Gould famously argued that the outcomes of biological evolution are often causally dependent on the precise series of changes that have occurred in the evolving lineages (Gould 1989; Blount et al. 2008, 2012; Zee et al. 2014). One possible basis for such historical contingency are epistatic interactions between mutations in determining fitness. Long-term evolution can be thought of as consisting of “one damned substitution after another,” to paraphrase the popular expression from 1909. If the effects of different mutations on fitness are completely independent of each other (i.e., if there is no epistasis for fitness), then later substitutions will not be causally dependent on earlier ones, and there will be no meaningful contingency. If, however, a mutation confers a benefit only in the presence of certain earlier mutations (i.e., if there is epistasis for fitness), then evolution will be historically contingent (Blount et al. 2018). In that case, the presence of a particular mutation determines the range of subsequent mutations that are likely to be favored. Epistasis for fitness means that even closely related populations might respond differently and unpredictably to similar selection pressures, depending on the substitutions they undergo, exactly as Gould envisaged (Blount et al. 2008; Wang et al. 2013; Harms and Thornton 2014; Spor et al. 2014; Zee et al. 2014; Shah et al. 2015; Phillips et al. 2016; Peng et al. 2018).

Historical contingency is difficult to examine because it requires disentangling the roles of chance and natural selection in determining evolutionary outcomes (Blount et al. 2018). One approach to this is to construct actual and alternative evolutionary pathways and determine the dependence of key phenotypes on the order in which mutations occur. This approach has been followed in several studies examining evolution of single genes, typically finding that epistasis creates a strong dependence of evolutionary outcome on the order in which mutations occur (Weinreich et al. 2006; Ogbunugafor and Hartl 2016; Starr et al. 2017). At the level of whole organisms, this approach is not generally feasible if large numbers of mutations have occurred. Instead, it is possible to use controlled laboratory experimental evolution to examine the evolutionary outcomes of replicated populations and determine statistically a signature of historical contingency (Blount et al. 2008, 2012; Zee et al. 2014).

Several experimental evolution studies have found that adaptive potential differs between different genotypes, often, at least in part, explained by differences in their initial fitness (Travisano et al. 1995; Barrick et al. 2010; Bedhomme et al. 2013; Kryazhimskiy et al. 2014; Perfeito et al. 2014; Wünsche et al. 2017; Johnson et al. 2019). The strong influence of fitness on mutation effect is supported by work that has directly measured the effect of adding specific mutations to diverse genetic backgrounds (Pearson et al. 2012; Wang et al. 2013; Kryazhimskiy et al. 2014; Wang et al. 2016). Nevertheless, specific genetic differences between founding strains can be influential (Blount et al. 2008, 2012; Zee et al. 2014). Indeed, historical contingency can affect replicate populations started from a common ancestor and evolved in a common environment (Collins and Bell 2004; Cooper and Lenski 2010; Wiser et al. 2013). In this case, contingency builds on chance differences in the origination and fixation of mutations. One example is the evolution of citrate utilization in an *Escherichia coli* long-term evolution experiment. In that work, one of 12 replicate populations evolved the ability to use citrate (Blount et al. 2008). Subsequent genetic analysis revealed that evolution of citrate utilization is rare because it depends on a series of prior mutational events (Quandt et al. 2014; Leon et al. 2018). Other examples of historical contingency have been inferred by finding genetically or phenotypically distinct evolutionary outcomes either dependent on early occurring mutations (Yedid and Bell 2002; Meyer et al. 2012) or from analysis of the total suite of changes occurring in evolved populations (Tenaillon et al. 2012).

**Fig. 1.**
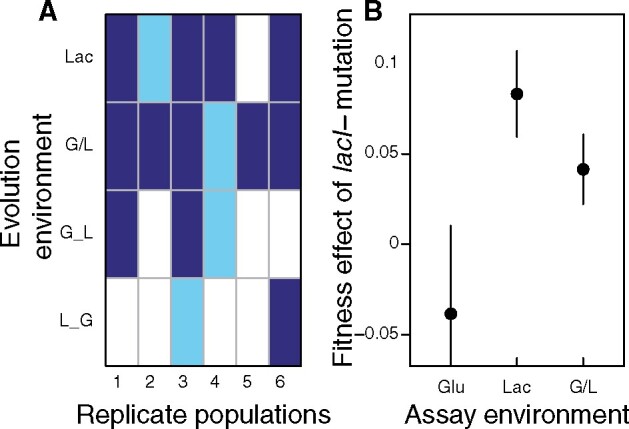
*lacI* effect and distribution in 8,000 generation evolved populations. (*A*) Rows represent each of four evolution environments (Lac [lactose], G/L [alternating daily between glucose and lactose], G_L [alternating every 2,000 generations between glucose and lactose, starting with glucose], L_G [alternating every 2,000 generations between glucose and lactose, starting with lactose]) and columns the six replicate populations evolved in each environment. Details are in Materials and Methods. Dark blue cells indicate fixation of the *lacI−* mutation, light blue cells indicate a mixed population with *lacI−* still segregating, and white cells indicate that *lacI−* was undetectable. Details of screening are in Materials and Methods. (*B*) Fitness effect of the *lacI−* mutation added to the ancestor and assessed in three evolution environments relative to the original ancestor (following method described in Materials and Methods). Symbols show mean and lines 95% CIs of at least three replicate competitions in each treatment.

We examine the basis of divergent evolution in *lacI*, the repressor of the *lac* operon genes that confer the ability to utilize lactose. Mutations in *lacI* fixed or rose to high frequency in 16 of 24 populations evolved for 8,000 generations in a long-term experiment that evolved populations of *E. coli* in environments containing combinations of glucose and lactose (Cooper and Lenski 2010; Satterwhite and Cooper 2015) (fig. 1*A* and supplementary fig. S1, Supplementary Material online). Absence of repressor mutations in the remaining eight populations was surprising because *lacI* mutations confer a large fitness benefit in the ancestor (fig. 1*B*) and occur at a high rate, due to the presence of a mutational hotspot (Farabaugh et al. 1978). Evolutionary simulations incorporating the measured *lacI−* mutation rate and fitness effect indicate that this divergence cannot be attributed to chance differences in mutation timing and success. When we added a *lacI−* mutation to strains isolated from populations that did not fix it, we found that it no longer confers any benefit, and, in fact, is often deleterious, indicating that it interacts negatively with previous substitutions. These results demonstrate that divergent adaptation in the *lac* operon is common and is due to effects contingent on previous adaptations.

## Results

### Mutations in *lacI* Occur in about Half of Evolved Populations

We previously found that loss-of-function mutations in the *lac* operon repressor, *lacI*, were common in nine of 12 populations of *E. coli* evolved for 2,000 generations in environments either containing lactose (Lac) only or alternating daily between lactose and glucose (G/L) (Quan et al. 2012). Here, we find that after extension for a further 6,000 generations of evolution, *lacI−* mutations had fixed or become common in two more of these populations and in five of 12 derived populations that alternated between glucose and lactose every 2,000 generations (denoted G_L and L_G where the first letter indicates the sugar initially present during selection; fig. 1*A*). The absence of detectable *lacI−* mutations in eight of the 24 populations was surprising because a mutational hotspot in *lacI* causes inactivating frameshift mutations to occur at a high rate and those mutations provide a benefit in lactose-containing environments that outweigh an associated cost in glucose (Farabaugh et al. 1978; fig. 1). Herafter, we denote evolved populations that did and did not fix a *lacI−* mutation *Ev^lacI−^ and Ev^lacI+^*, respectively.

A possible explanation for the failure of *lacI−* mutations to fix after 8,000 generations in multiple populations is that an earlier occurring substitution(s) reduced either the rate at which *lacI−* mutations occur or the benefit they confer when they do arise. We refer to this possibility as the contingency hypothesis. An alternative, noncontingent explanation is possible, however. We examine these hypotheses below.

### Simulations Predict More *lacI−* Substitutions than Observed

**Fig. 2.**
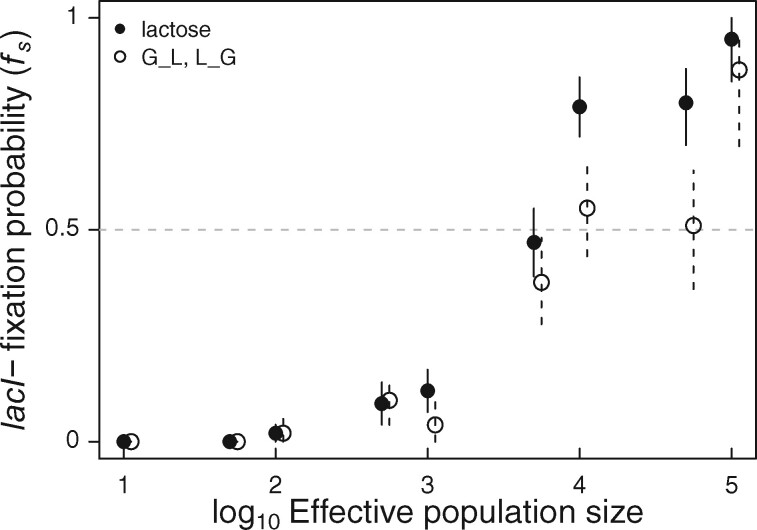
Simulated frequency of *lacI−* substitution (*fs*). Simulations were modeled in Lac and extrapolated to apply to G_L and L_G 2,000-generation fluctuating environments as detailed in Materials and Methods. Symbols indicate the proportion of replicate simulations (*n *=* *100 except at *I*_e_* *=* *5 × 10^4^ [*n *=* *50] and *N*_e_* *=* *10^5^ [*n *=* *20]) in which *lacI−* fixed. Error bars are 95% CI. Means and errors were calculated using 1,000 boot-strapped samples. In the Lac treatment, populations were simulated for 8,000 generations. For G_L and L_G 2,000 generation fluctuating environments, the same simulated populations were sampled at 2,000 generations and frequency of *lacI−* substitution (*fs*) was calculated as detailed in Materials and Methods. The dashed line indicates the overall frequency of *lacI−* fixation at 8,000 generations among 24 experimentally evolved populations. Note that the two treatments are plotted with a small offset to avoid overplotting.

An alternative to the contingency hypothesis is that *lacI−* mutations, although beneficial, have simply not occurred yet, or have occurred but have not yet had the time to reach high frequency. To evaluate this possibility, we simulated the evolution of populations with *lacI−* mutations available alongside a background pool of other beneficial and deleterious mutations (fig. 2). We used an individual-based model, incorporating estimates of mutation rates and effects, and allowing for competition between mutations (see Materials and Methods for details). Our results show that, as populations become larger, the frequency of substitution (*f*_s_) for a *lacI−* mutation rises quickly. With an effective population size of 10^5^ individuals, *lacI−* fixed in most simulated populations (*f*_s_ = 91.5% combining Lac, G_L and L_G selection regimes). The observed frequency in experimental populations was only *f*_s_ = 12/24 = 50% (95% CI: 31.4–68.6%) despite the fact that they were much larger (*N*_e_ = 3.3 × 10^7^) (Cooper and Lenski 2010). This difference is statistically significant (binomial test: *P *<* *0.001). Thus, stochastic variation in mutation timing and competition cannot explain the high number of experimental populations retaining the ancestral *lacI* allele.

### Evolved Clones Have Similar *lacI−* Mutation Rates to Ancestor

**Fig. 3.**
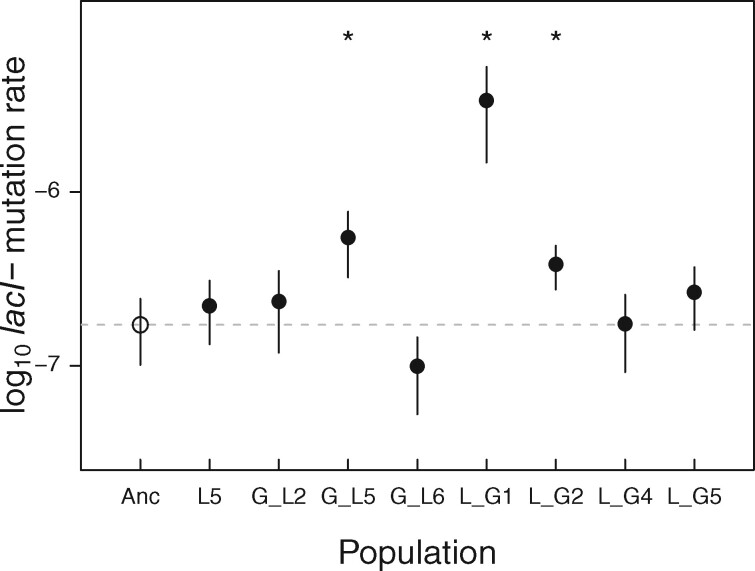
Mutation rate to *lacI−* for 8,000 generation *lacI+*_ev_ populations. Closed symbols indicate evolved populations and the open circle (and dashed line) indicates the mutation rate in the ancestor. Mean per locus mutation rates are calculated from *n *≥* *10 replicates, and errors are 95% CIs. Asterisks indicate populations with a *lacI* mutation rate significantly different than the ancestor (*t*-test *P *<* *0.05).

A second hypothesis for *lacI−* mutations failing to fix in some populations is that their mutation rate was lower in those populations. Indeed, mutation at the *lacI* hotspot involves a strand slippage mechanism that might be affected by DNA topology, which is a common target of selection in an experiment closely related to this one (Blount et al. 2008, 2012; Zee et al. 2014). To the extent that a potential change in *lacI−* mutation rate depends on previously substituted mutations, this possibility represents an example of contingency. We estimate the ancestral *lacI−* mutation rate at 1.72 ± 0.7 × 10^−7^ per cell per generation (95% CI), which is consistent with a previous estimate (Schaaper et al. 1986). If Ev^*lacI*^^+^ populations evolved lower *lacI−* mutation rates, expected fixation times would be increased. In fact, five of eight clones isolated from Ev^*lacI*^^+^ populations had *lacI−* mutation rates indistinguishable from the ancestor (fig. 3). The remaining clones had an elevated *lacI−* mutation rate. In the case of the L_G1 population, this elevation was substantial, presumably due to the presence of a 27-bp deletion in the *mutS* gene, which is likely to result in a general increase in the genomic mutation rate (supplementary table S1, Supplementary Material online).

In summary, changes in mutation rate to *lacI−* do not explain why it has not fixed in so many evolved populations. We note, however, that this analysis does not exclude the possibility that populations might have evolved in a way causing beneficial mutations at loci other than *lacI* to occur at higher rates and that these mutations might outcompete mutations occurring at *lacI* (Gerrish and Lenski 1998). We consider this possibility unlikely, however, because it would delay but not prevent *lacI−* mutation substitutions. Moreover, results presented below support an alternative mechanism affecting *lacI−* fixation.

### Fitness Effect of *lacI−* Is Lower in Ev^*lacI*^^+^ Populations

To directly test for a change in the selective benefit of *lacI−* mutations as posited by the contingency hypothesis, we performed allelic replacement experiments to measure the fitness effect of *lacI−* mutations in clones isolated from all evolved populations. If the *lacI−* mutation was prevented from fixing due to epistasis, we expected it to be less beneficial, or even deleterious, in Ev^*lacI*^^+^ populations.

**Fig. 4.**
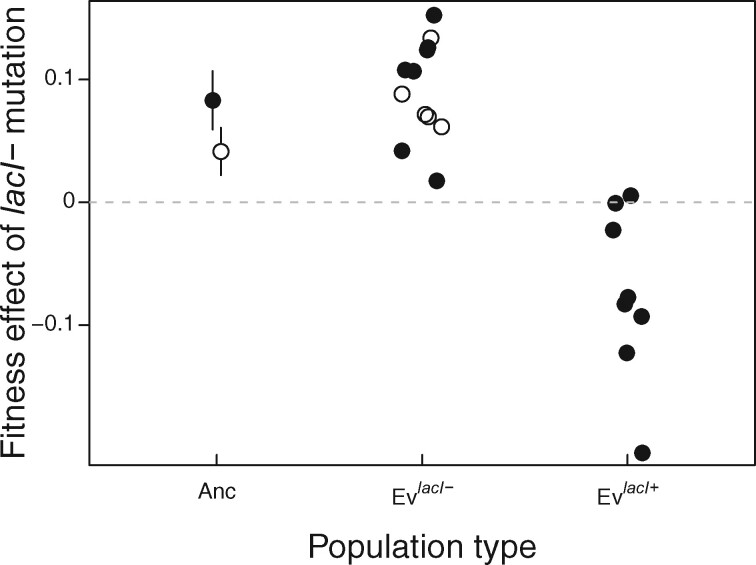
Fitness of *lacI−* in ancestor and 8,000 generation evolved populations. Solid symbols indicate the fitness measurements for the ancestor and for clones from populations evolved in lactose, G_L and L_G environments, which were measured in lactose. Hollow symbols indicate the ancestor and clones isolated from populations evolved in the G/L environment, which were measured in the G/L environment. Symbols indicate mean fitness estimates (*n* ≥ 3). Errors shown for ancestor are 95% CIs.

We found that the *lacI−* mutation was beneficial in the 12 populations in which it fixed (fig. 4). Among clones isolated from populations selected in Lac, G_L, and L_G environments, the grand mean fitness effect of the *lacI−* mutation measured in the lactose environment is 9.66% (±2.29% [95% CI]), which is not significantly different from its effect in the ancestor (two-tailed *t*-test, *P *=* *0.53). Among clones isolated from the G/L environment, the *lacI−* mutation conferred a significantly greater benefit than it did in the ancestor measured in that environment (8.50% vs. 4.05%, two-tailed *t*-test: *P *=* *0.02). By contrast, the *lacI−* mutation conferred no benefit or was deleterious when introduced into clones isolated from the eight Ev^*lacI*^^+^ populations, imposing a mean cost of 7% (±4.81% [95% CI]) when measured in lactose (compared with its effect in the ancestor, two-tailed *t*-test: *P *<* *0.001) (fig. 4). These results reveal the presence of strong negative epistatic interactions between the *lacI−* mutation and one or more of the mutations that accumulated in the Ev^*lacI*^^+^ populations, in agreement with the contingency hypothesis.

### Ev^*lacI*^^+^ Populations Have an Alternative Mechanism of Reducing Lag Time

**Fig. 5.**
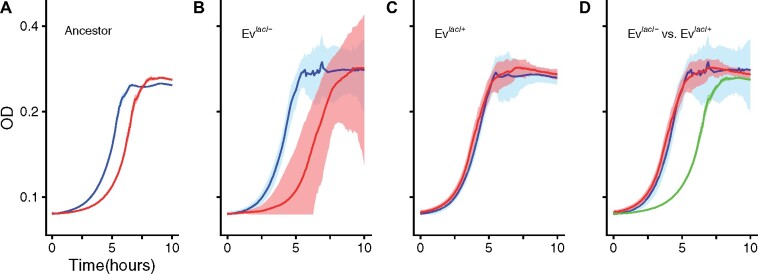
Effect of *lacI−* mutation on growth dynamics of the ancestor and evolved clones. Red and blue lines indicate strains(s) with functional and nonfunctional *lacI* alleles, respectively. The green line in (*D*) indicates the ancestral *lacI+* strain, as plotted in panel (*A*), and is included for reference. Shaded regions indicate SD (*n* ≥ 3). All growth curves performed in the lactose environment. (*A*) Ancestor with and without the *lacI−* mutation. The mutation significantly decreases lag time in the lactose assay environment. (*B*) Clones from Ev^*lacI−*^ populations. The *lacI−* mutation also decreases lag time in these populations. (*C*) Clones from Ev^*lacI*^^+^ populations. The *lacI−* mutation confers a small cost when added to these clones. (*D*) Original evolved clones from Ev^*lacI−*^ and Ev^*lacI*^^+^ populations. The shortened lag time dependent on the *lacI−* mutation in Ev^*lacI−*^ populations has been achieved through a different mutational mechanism in Ev^*lacI*^^+^ populations. Lines indicate the mean of replicate growth curves for a single strain (*A*) or a grand mean of growth curves estimated for the multiple clones in the noted evolutionary group (*B–D*).

To examine the basis of the different fitness effects of the *lacI−* mutation, we compared its effect on growth dynamics in the ancestor, and in Ev^*lacI−*^ and Ev^*lacI*^^+^ clones. In the ancestor, the most striking effect of the *lacI−* mutation was a decrease in the time taken for the population to resume growth following transfer to fresh medium (i.e., its lag time) from 5.00 to 3.87 h (*t*-test: *P *<* *0.001; fig. 5*A* and supplementary fig. S2, Supplementary Material online). This effect was also seen among Ev^*lacI−*^ clones (Ev^*lacI−*^ clones: lag = 2.96 h; *lacI+* revertants: 5.60 h; paired *t*-test: *P *<* *0.001; fig. 5*B* and supplementary fig. S2, Supplementary Material online). By contrast, the *lacI−* mutation increased lag time when added to Ev^*lacI*^^+^ clones (Ev^*lacI*^^+^ clones: lag = 2.96 h; *lacI−* derivatives: 3.11 h; paired *t*-test: *P *=* *0.03; fig. 5*C* and supplementary fig. S2, Supplementary Material online). We found good agreement between direct competitions between *lacI+/−* strain pairs and virtual competitions based on growth curve parameters, indicating that growth parameters are meaningful measures of fitness components (supplementary fig. S2, Supplementary Material online).

The absence of *lacI−* mutation benefit may be because Ev^*lacI*^^+^ clones had substituted an alternative means of shortening lag time such that the effect of the *lacI−* mutation has become redundant. To test this possibility, we compared the lag times of Ev^*lacI*^^+^ and Ev^*lacI−*^ clones. We found that lag times were indistinguishable, consistent with Ev^*lacI*^^+^ populations having evolved an alternative means of shortening lag time (two-tailed *t*-test *P *=* *0.96; fig. 5*D*).

### Ev^*lacI*^^+^ Populations Have Increased Sensitivity to a *lac* Operon Inducer

As a first step in examining the basis of the reduction of lag time among Ev^*lacI*^^+^ populations, we measured changes in the expression of *lac* genes in environments with increasing concentrations of IPTG, a synthetic inducer. We found that clones from all Ev^*lacI*^^+^ populations were more sensitive in responding to IPTG, reaching half maximum expression of LacZ, a gene product controlled by the LacI repressor, at a lower concentration of IPTG than the ancestor (supplementary fig. S4, Supplementary Material online). Qualitatively similar results were seen when we used an alternative inducer, TMG, and compared expression of a P_*lac*_-*gfp* reporter at 4 and 6 h following induction (supplementary fig. S5, Supplementary Material online). These findings are consistent with a higher sensitivity to IPTG and TMG inducers in the Ev^*lacI*^^+^ strains.

An increase in inducer sensitivity could cause reduced lag times in Ev^*lacI*^^+^ populations by allowing them to more quickly express *lac* genes following transfer to a fresh lactose supplemented environment. One mechanism through which this could occur is if basal *lac* expression was higher in Ev^*lacI*^^+^ populations, which increases the probability that a cell will turn on *lac* operon expression and resume growth (Chu and Barnes 2016). However, in both the IPTG and TMG induction data experiments, basal *lac* expression appeared unchanged in Ev^*lacI*^^+^ populations relative to the ancestor. We also considered the possibility of some change in the nature of lactose uptake. None of the evolved strains had mutations in the LacY permease protein so, to test the possibility that some other inducer import mechanism had evolved, we determined the reliance of *lac* induction on the LacY permease. To do this, we introduced a *lacY* loss-of-function mutation into the ancestor and the Lac5 Ev^*lacI*^^+^ strain. (This strain evolved a strong antagonistic interaction with the *lacI−* mutation, see below.) We found that increased sensitivity of *lac* induction, in response to both lactose and TMG, was completely dependent on LacY (supplementary fig. S6, Supplementary Material online). We also found that TMG, but not lactose, was able to weakly induce *lac* genes independent of LacY, but that ability was not different between the ancestor and the Lac5 strain.

### Reduced Benefit of *lacI* Mutations Is Caused by Mutations in *uspB*

**Fig. 6.**
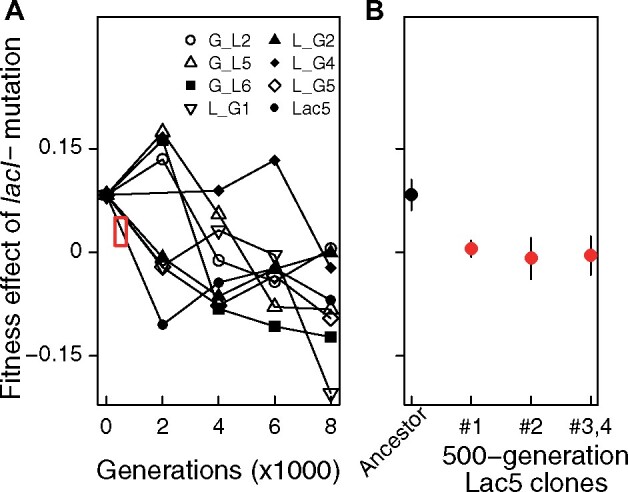
Fitness effect of the *lacI−* mutation introduced into Ev^*lacI*^^+^ clones. (*A*) Symbols indicate the effect of adding the *lacI−* mutation into clones from each Ev^*lacI*^^+^ population isolated at indicated time points. Lines connect clones isolated from the same population at different points in time. The red rectangle indicates the population and 500 generation time point that clones were isolated from to analyze in more detail (shown in *B*). We were unable to transfer the *lacI−* mutation into a clone isolated at 2,000 generations from one Ev^lacI−^ population. The mutation effect trajectory of this population connects the ancestor and the 4,000 generation time point directly. (*B*) The effect of the *lacI−* mutation in the ancestor (black symbol) and clones from the 500 generation time point of the Lac5 population (red symbols). Clones 3 and 4 had the same genotype so are grouped here (table 1). Mean and 95% CI of replicate fitness estimates are shown (*n* = 3).

To examine the nature of the mutations that interact with *lacI−* to reduce its benefit, we estimated the fitness effect of a *lacI−* mutation introduced into clones isolated at intervals along the evolutionary path of the eight Ev^*lacI*^^+^ populations (fig. 6*A*). We reasoned that steep declines in *lacI* mutation effect would indicate the substitution of a negatively interacting mutation in the population. Among the tested populations, the steepest change was seen in the Lac5 population. Focusing on the beginning part of the evolution of this population, the effect of the *lacI−* mutation declined from conferring an 8.3% benefit in the ancestor to a mean cost of 0.9% among four independent clones isolated from this population at 500 generations of selection (fig. 6*B*). To determine the genetic basis of this change, the 500 generation evolved clones were sequenced and mutational changes relative to their ancestor identified (table 1). All four clones shared an IS*150* insertion occurring 24-bp upstream of the *uspB* gene. The same mutation is present in clones isolated from this population after 4,000 and 8,000 generations of evolution.

**Table 1.** Mutations in 500 Generation Clones of the *Lac*5 Population.

	Clone			
Gene	1	2	3	4
*uspB* ^a^←::IS*150*→*uspA*	–24/–367^b^ bp	–24/–367 bp	–24/–367 bp	–24/–367 bp
*rbs*		Δ5,943 bp	Δ7,590 bp	Δ7,590 bp

a Also known as *yhiO*.

b Numbers indicate position of the insertion relative to the start of the flanking genes.

To determine the interaction between *lacI−* and *uspB* mutations, we focused on a Lac5 8,000 generation evolved clone. The *lacI−* mutation confers a cost in this clone measured in the lactose environment (Lac5 *lacI−* vs. Lac5: relative fitness = 0.89 ± 0.09 [95% CI], *P *=* *0.03). When we reverted the evolved *uspB* allele, the *lacI−* mutation conferred a 26% benefit, comparable with its effect in Ev^*lacI−*^ clones and greater than its effect in the ancestor (Lac5 *uspB*^Anc^*lacI−* vs. Lac5 *uspB*^Anc^: relative fitness = 1.26 ± 0.09 [95% CI], *P *=* *0.006). This result identifies the *uspB* mutation as being one cause of the historical contingency we see, acting to reduce the benefit conferred by *lacI−*. We note, however, that the situation is complex and might depend on higher order interactions. Insertion mutations upstream or in *uspB* are present in all of seven sequenced clones isolated from Ev^*lacI*^^+^ populations, but also in clones isolated from four Ev^*lacI−*^ population (G_L3, G/L5, L_G6, and Lac1) (supplementary tables S1 and S2, Supplementary Material online). This pattern indicates that clones without the *lacI−* mutation are significantly more likely to have the *uspB* mutation, consistent with a negative genetic interaction (one-tailed Fisher’s exact test: *P *=* *0.007). Nevertheless, that *uspB* and *lacI−* are sometimes found together suggests that other mutations might influence the relationship between them.

### Divergent *lac* Regulation Strategies Do Not Promote Further Genetic Divergence

To test if the historical contingency influencing the effect of the *lacI* mutation extends to promoting continued divergence of Ev^*lacI*^^+^ and Ev^*lacI−*^ populations along different evolutionary paths, we used Dice’s coefficient of similarity (s) to compare the sets of mutations found among each population group (supplementary tables S1 and S2, Supplementary Material online). This metric is bounded between 0, indicating completely dissimilar mutation sets, and 1, indicating identical mutation sets. We found no signal of higher mutation parallelism within compared with between populations that substitute different *lacI* strategies. On average, Ev^*lacI*^^+^ clones were more similar to Ev^*lacI−*^ clones than to each other (within Ev^*lacI*^^+^, Dice’s *s* = 0.045; between Ev^*lacI*^^+/−^, *s* = 0.063; within Ev^*lacI−*^, *s* = 0.073) (supplementary fig. S7*A*, Supplementary Material online). Moreover, no similarity estimate was significantly different to a null expectation based on randomly assigning sequenced clones to the Ev^*lacI*^^+^ and Ev^*lacI−*^ group labels. A limitation to this analysis is that it groups populations that were selected in different environments. Although it would be ideal to consider the effect of *lacI−* on subsequent mutation accumulation separately for each selection environment, only one of four environments contains multiple populations of each *lacI* type, limiting our ability to distinguish repeated from random effects. We note, however, that, with one exception, clones from the different selection environments did not detectably differ in the sets of mutations they accumulated (supplementary fig. S7*B*, Supplementary Material online).

## Discussion

We found that the absence of potentially beneficial *lacI* mutations in Ev^*lacI*^^+^ populations was due to epistasis and is, therefore, a case of historical contingency. In other words, populations followed at least two distinct evolutionary paths contingent on the presence of some earlier mutational change. This early mutation caused the fitness effect of subsequent *lacI−* mutations to be neutral or deleterious in all Ev^*lacI*^^+^ populations, instead of beneficial as in the ancestor and Ev^*lacI−*^ populations. We found no evidence to support alternative hypotheses of insufficient time or changes in mutation rate explaining the absence of *lacI−* mutations.

In the context of replicate evolved populations started from a common ancestor, as examined here, historical contingency stems from negative genetic interactions between early mutations and potentially beneficial mutations. At the physiological level, these interactions reflect that otherwise beneficial mutations are either redundant to, or interact antagonistically with, earlier substituting mutations. Redundancy could occur if there were two alternative mutational targets that cause the same phenotypic change. For example, mutations affecting either repressor proteins or operator binding sites (Quan et al. 2012). Antagonism could reflect, for example, alternative adaptive strategies that share some metabolite such that the evolution of one strategy changes the cellular physiological environment in a way that means the second is no longer beneficial (reviewed in De Visser et al. [2011]). Whereas redundancy allows for convergence in adaptive traits, even as populations might diverge genetically, antagonism between adaptive mechanisms predicts eventual phenotypic divergence as new mutations come to depend on the different cell environments caused by earlier ones (Szamecz et al. 2014).

We find that the shortened lag time conferred by *lacI−* mutations in the Ev^*lacI−*^ populations is matched in Ev^*lacI*^^+^ populations, consistent with Ev^*lacI*^^+^ populations substituting some alternative mutation(s) that makes *lacI−* redundant. This possibility is also supported by our findings that Ev^*lacI*^^+^ and Ev^*lacI−*^ populations had indistinguishable growth curves, that Ev^*lacI*^^+^ populations increased in their sensitivity to *lac* operon induction, and that the overall set of substituted mutations did not differ between the population groups. Indeed, it seems that “evolutionary routes are many, but the destinations are limited.” (Blount et al. 2008, 2012; Zee et al. 2014). A purely redundant mechanism of interaction does not, however, easily explain our finding that adding *lacI−* decreases fitness in five of eight Ev^*lacI*^^+^ strains more than can be explained by only the cost of constitutive *lac* expression. These high costs indicate some direct antagonism between *lacI−* and alternative adaptations. To the extent that this antagonism reflects a distinct mechanistic basis of adaptation, albeit with similar immediate phenotypic effects, it might eventually lead to diverging selective opportunities and, therefore, evolutionary paths.

The mechanism by which the *lacI−* mutation shortens lag time likely depends on the lack of functional repressor causing cells to produce higher levels of *lac* gene products through stationary phase so that they can more quickly import and catabolize lactose in fresh medium (Quan et al. 2012; Chu and Barnes 2016). The most obvious candidate for a mutation interacting with *lacI−*, therefore, would be one causing the same higher *lac* activity. We did not find any candidate mutations in the canonical *lac* regulon of Ev^*lacI*^^+^ clones, and, indeed, we did not detect any increase in basal *lac* expression among these clones. However, we did identify an IS*150* insertion mutation occurring upstream of *uspB* as interacting with *lacI* in at least one population. The insertion occurs just upstream of *uspB* between the promoter and the start of the gene. It therefore seems likely to reduce or eliminate that gene’s expression, though IS*150* has an outward-facing promoter and could also upregulate *uspB* (Schwartz et al. 1988). In either case, a link between UspB and lactose metabolism is unclear. UspB is an integral membrane protein that is up-regulated in stressful conditions, including starvation, and might play a role in mediating changes in membrane composition either directly or as a result of disrupted protein–protein interactions (Farewell et al. 1998; Liu et al. 2019). In principle, such changes could affect stationary phase membrane composition to facilitate lactose uptake although lactose induction of *lac* expression still depended on the LacY permease so any change in uptake must have been minor (supplementary fig. S6, Supplementary Material online). We did not rule out the possibility that *uspB* interacts indirectly with lactose metabolism, though the benefit of *lacI−* was lost in strains containing only *uspB* and one other mutation, a deletion of the *rbs* operon. The *rbs* deletion mutation occurred in almost all of our evolved populations, regardless of their *lacI* status, so it seems likely the interaction is, in fact, direct. Finally, we note that although the *uspB* mutation was found in all Ev^*lacI*^^+^ clones, it was also found in one Ev^*lacI−*^ population, indicating that the interaction between *uspB* and *lacI* mutations might be influenced by other mutations.

Both Ev^*lacI*^^+^ and Ev^*lacI−*^ populations are found in three of the four lactose-containing environments we consider, but at different frequencies. The ratio of *lacI−* absence to fixation (7:3) is substantially higher among the 2,000 generation fluctuating G_L and L_G selected populations than among those selected in the daily alternating G/L environment (0:5) (Fisher’s exact test: *P *=* *0.026), even though populations spent the same total time in each environmental component. The *lacI−* mutation was also less likely to fix among G_L and L_G populations than among Lac populations, although this difference was not significant (Fisher’s exact test: *P *=* *0.119). One explanation for these differences is that mutations selected in the glucose portion of the selection regime can interact negatively with *lacI−*, decreasing its benefit in lactose. Such mutations would not be expected to reach high frequency in the G/L daily fluctuating environments where they would compete directly with *lacI−* mutations for fixation.

How the history of a population affects its future evolution remains a major question in evolutionary biology (reviewed in Blount et al. [2018]). Does natural selection generally converge on high-fitness genotypes, such that evolutionary outcomes, even if not paths, are deterministic, or do antagonistic interactions cause chance differences in mutation occurrence and success to be built on, causing evolutionary outcomes to be contingent, and, therefore, unpredictable? Contingency leading to divergent fitness outcomes has been described in several controlled laboratory evolution experiments, even when comparing populations started from a common ancestor, and therefore having relatively few differences among which contingency can arise (Collins and Bell 2004; Blount et al. 2008; Beaumont et al. 2009; Cooper and Lenski 2010; Wiser et al. 2013). Divergence is commonly seen at the genetic level in these kinds of experiments, though the basis of differences is not usually examined. For example, whether distinct mutational pathways are likely to be equivalent or to affect available subsequent adaptive paths, and therefore likely to lead to eventual fitness differences. One example of the difficulty in evaluating this possibility comes from an experiment demonstrating a reproducibly different adaptive potential of genotypes differing crucially in mutations causing different residues in a gene affecting DNA supercoiling (Woods et al. 2011).

In summary, we describe an example of historical contingency affecting selection of *lac* regulation. Whereas approximately half of a set of replicate populations selected in lactose-containing environments quickly substituted a mutation in *lacI* that provided a benefit by decreasing lag time, remaining populations substituted other mutations that interacted negatively with *lacI* causing them to follow alternative evolutionary paths. At the point in our evolution experiment that was considered here, these paths are marked by distinct genetic, but not fitness, outcomes.

## Materials and Methods

### Bacterial Strains and Growth Conditions

Bacterial strains were isolated from populations evolved for 8,000 generations as part of a long-term evolution experiment. This experiment evolved replicate populations in seven different environmental treatments, four of which are considered here: lactose only (Lac), a combination of glucose and lactose fluctuating daily (G/L), or the same combination fluctuating every 2,000 generations with one treatment starting with glucose (G_L) and another with lactose (L_G) (Satterwhite and Cooper 2015) (supplementary fig. S1, Supplementary Material online). Evolution environments comprised these sugars added to base Davis–Mignoli (DM) medium at a concentration of 175 μg/ml (glucose) or 210 μg/ml (lactose). These concentrations support approximately equal densities of stationary phase bacteria (∼3.5 × 10^8^ cfu/ml) (Cooper and Lenski 2010). Populations were propagated in 1 ml of medium in 96 × 2 ml well blocks for 8,000 generations, using a daily 1:100 serial transfer. Each population was initially homogeneous, such that de novo mutation was the only source of genetic variation. Samples were frozen at *−*80 °C, with glycerol as a cryoprotectant, every 500 generations. Six replicate populations were evolved in each environment, with three started from REL606 and three from REL607, a spontaneous Ara+ derivative (Lenski et al. 1991). The different *ara* markers allow strains to be differentiated by plating on tetrazolium-arabinose plates, on which *ara+* strains form white colonies and *ara−* red colonies. The Ara marker does not affect fitness measurements in any of the assay environments (Cooper and Lenski 2010). Lysogeny broth (LB) was used for nonselective culturing, whereas the respective evolution environment of each clone was used for fitness assays and growth measurements.

### Identification of *lacI* Mutations

To determine fixation of *lacI* mutations within evolved populations, we plated 8,000-generation population samples (1,000–3,000 cells) on TGX indicator medium (Blount et al. 2008, 2012; Schenk et al. 2012; Zee et al. 2014). This medium consists of 10 g/l tryptone, 2.5 g/l sodium chloride, 5 g/l glucose, and 30 mg/ml of the LacZ substrate 5-bromo-4-chloro-3-indolyl-beta-d-galactopyranoside (X-gal). Colony color on this medium indicates the level of LacZ activity in the absence of any recognized inducer, and thus distinguishes between ancestral repressed (white colonies) and mutant derepressed (blue colonies) *lac* expressing strains. Four populations had a mix of blue and white colonies and were not considered here. Strains were isolated from populations having >95% blue colonies and sequenced to confirm that derepression was due to a mutation in the *lacI* repressor. Evolved strains from populations that fixed a *lacI* mutation are denoted as Ev^*lacI−*^ and those from populations that maintained the ancestral allele are Ev^*lacI*^^+^.

### Strain Construction

To determine the fitness effect of *lacI−* mutations in evolved strains**, **we reverted them using the suicide plasmid pDS132::*lacI+*, as described previously (Satterwhite and Cooper 2015). Potential engineered revertants were screened for expected *lacI+* phenotypes first on TGX medium. White colonies were streaked onto minimal medium supplemented with P-Gal. Clones that reverted to *lacI+* cannot grow on this medium (Nghiem et al. 1988). Clones isolated from populations that retained the ancestral *lacI* allele were converted to *lacI−* by isolating spontaneous mutants selected on minimal medium supplemented with P-Gal as the only potential carbon source and with X-gal as a means to increase visual contrast of small colonies, making them easier to detect. In all cases, mutant isolation was confirmed by sequencing of the *lacI* gene. This also allowed us to confirm that selected *lacI−* strains had the same 4-base insertion/deletion mutations that dominated the mutations that fixed in the evolution experiment (Quan et al. 2012).

We used a combined CRISPR-recombineering approach to replace the evolved *uspB* allele (*uspB::*IS*150*) with the ancestral allele (Jiang et al. 2015). A *uspB* targeting sgRNA pTarget plasmid (pTarget-*uspB*) was constructed by using the NEBuilder HiFi DNA Assembly kit to assemble DNA fragments containing a donor region of the ancestral *uspB* locus, the pTarget vector backbone including a *uspB* N20 sequence, and two linking oligonucleotides (details in supplementary table S1, Supplementary Material online). The *uspB* donor DNA encoded by the resulting plasmid does not alter the N20 target sequence in the edited strain. To prevent persistent editing after DNA repair, we modified the procedure used by Jiang et al. (2015) by simultaneously treating cells with arabinose and lactose for 1.5 h following transformation of pCas containing cells with pTarget-*uspB*. This combination activates the red recombination system and cuts the pTarget plasmid, causing it to be lost from cells. Transformants were plated on kanamycin plates to select pCas carrying cells and then grown at 42 **°C** to select for loss of that plasmid. The candidate edited cells were screened and confirmed by colony PCR.

### Mutation Rate Estimates

The rate of mutation to constitutive *lac* expression was estimated using a fluctuation test. For each clone, the freezer stock was inoculated into LB, grown overnight at 37 °C, and then diluted 1:1,000 into ten fresh 1 ml LB cultures. After overnight growth, a 100-μl sample from each replicate population was plated onto DM agar supplemented with X-gal and P-gal. A diluted sample was also plated onto LB agar plates to estimate total cell density. Plates were incubated at 37 °C for 48 h prior to counting.

To assess the plating efficiency of *lacI−* mutants on the X-gal + P-Gal selection plates, we compared the number of colonies growing when a small number of *lacI−* cells were plated by themselves on to permissive LB medium and when they were plated onto P-gal medium with an excess of a REL606 strain that had a *lac* operon deletion that made it unable to mutate to grow on P-Gal. The plating efficiency of *lacI−* cells was calculated as *p*_eff_ = *m*_c_/*m*_e_, where *m*_c_ is the number of *lacI−* mutants able to form visible colonies on P-Gal in combination with the deletion strain, and *m*_e_ is the number of mutants that form colonies when plated alone onto LB. Colony counts in the fluctuation test were multiplied by 1/*p*_eff_ to obtain corrected mutation counts. Following this correction, mutation rate analysis was carried out using the bz-rates estimator (http://www.lcqb.upmc.fr/bzrates) (Gillet-Markowska et al. 2015).

### Individual-Based Simulations

The frequency of substitution (*f*_s_) for a *lacI−* mutation is a function of mutation rate, population size, and mutation dynamics. These dynamics, such as clonal interference and hitchhiking, can significantly affect fixation times. To estimate the expected fixation time for *lacI−* mutants in the long-term evolution experiment, we conducted individual-based simulations using a Wright–Fisher regime with a genome-wide mutation rate of *U *=* *7 × 10^−4^, the mean genomic rate as calculated from relevant estimates (Wielgoss et al. 2011; Lee et al. 2012; Long et al. 2016), and a *lacI*+ to *lacI−* mutation rate of 1.72 × 10^−7^ (measured in the ancestor, see below). Competing background mutations were divided into three deleterious and three beneficial classes, each comprising a different proportion of occurring mutations (supplementary table S2, Supplementary Material online). Each background mutation occurs at a new site and acts on fitness independently of other mutations in a simulated individual. Mutations to *lacI−* conferred a fitness increase as measured in this work in the ancestor (8.31% in Lac, 4.05% in G/L; fig. 1*B*). The effective population size prevailing in the evolution experiment (*N *=* *3.3 × 10^7^) (Cooper and Lenski 2010) was too large for us to replicate simulations in a reasonable time frame. We therefore simulated population sizes up to 10^5^. Fixation times are expected to be decreased at larger population sizes, therefore results at *N *=* *10^5^ provide a conservative estimate for the probability of a *lacI−* mutation fixing in the experimental populations. Populations were allowed to evolve for 8,000 generations or until the *lacI−* mutation fixed (frequency above 95%). For each population size, we simulated 100 replicate populations and recorded the proportion of these replicates that fixed the *lacI−* mutation. We then bootstrapped the simulation results 1,000 times to estimate the 95% CI for *f*_s_. For the 2,000-generation fluctuating environments, we simulated only the two 2,000 generations of evolution in lactose, and calculated *f*_s_ as *f*_s_ = *f*_2k_ + *f*_2k_(1 − *f*_2k_), where *f*_2k_ is the frequency of *lacI−* fixation during the first period of 2,000-generations of lactose selection. Assuming that *lacI−* mutations that did not fix during lactose selection periods were lost during any subsequent period of glucose selection, where they were costly, the second term gives the number of those populations expected to fix *lacI−* in the second period of lactose selection.

We did not simulate evolution in the G/L environment, as this requires estimation of the correlated effects of background mutations across glucose and lactose environments, which are ignored in the more slowly fluctuating 2,000 generation environments.

### Fitness Assays

The relative fitness of a given strain was assayed relative to a reference with the opposite Lac (i.e., *lacI+* vs. *lacI−*) or Ara (i.e., *ara+* vs. *ara−*) marker. Fitness assays were carried out in identical conditions to a tested strain’s evolution environment. Prior to each assay, competitors were independently preconditioned to the competition environment for 1 day, except for the G/L environment, which was preconditioned over 2 days. For the G/L environment, cultures were diluted 100-fold from glucose to lactose for day two (for both preconditioning and competition steps). Following preconditioning, competitors were mixed at a 1:1 volume ratio and transferred with a 100-fold dilution into the competition environment. A sample was immediately plated on indicator agar (TGX or TA) in order to determine the starting frequency of the two competitors. At the end of the competition, samples were again plated on indicator agar. Absolute fitness of competitor, *a*, was calculated as *w*_a_ = ln(100^*d*^×(*N*_a_(*f*)/*N*_a_(*i*)), where *d* is the number of competition days, and *N* is the number of colony-forming units at the initial (*i*) and final (*f*) time points. The relative fitness of competitor *a* relative to competitor *b* is then *w*_a/b_ = *w*_a_/*w*_b_, and the selective advantage of *a* over *b* is *s* = *w_a/b_*−1.

### Growth Rate Estimates and Virtual Competitions

Each clone was inoculated from a freezer stock into 1 ml of LB media and grown overnight. The following day, 1 μl of each culture was transferred into 1 ml of DM supplemented with 210 μg/ml lactose and incubated at 37 °C for 24 h. On day three, 2 μl of the preconditioned culture was transferred into 198 μl of the same media in a 96-well polystyrene plate. This plate was incubated in a VersaMax microplate reader (Molecular Dynamics, CA) and grown at 37 °C until cells reached stationary phase. The culture’s optical density at 450 nm (*OD*450) was measured every 5 min during growth. Growth curves were analyzed using an extension of the logistic model (Baranyi and Roberts 1994; Ram et al. 2019) as implemented at: https://multi-choice-comparison.shinyapps.io/growth_curves/. To check that estimated growth parameters were meaningful estimates of fitness components, we implemented a double-strain Baranyi–Roberts model to perform virtual competitions as mediated only through growth parameters separately estimated for each competitor (Ram et al. 2019). Virtual competition results were compared with direct competition fitness estimates. Briefly, virtual competitions model a common nutrient pool that is independently used for growth by two competitors depending on their measured growth parameters. The change in the density of each competitor was used to estimate a virtual relative fitness.

### Genome Sequencing

Genomic DNA was isolated and purified using the Wizard Genomic DNA Purification Kit (Promega) following the protocol for Gram-negative bacteria at one-third volume. Double-stranded DNA was quantified using SYBR Green I Nucleic Acid Stain (Invitrogen) in a SpectraMax M5 Fluorescence Microplate Reader (Molecular Devices). Libraries were created following the Nextera XT DNA Library Prep Kit protocol, at one-quarter volume, with Nextera XT Index Kit v2 adapters (Illumina). Libraries were individually quantified using the Qubit dsDNA High Sensitivity Assay with a Qubit 2.0 Flourometer (ThermoFisher) and fragment size determined using the Agilent 2100 BioAnalyzer with a High Sensitivity DNA Analysis Kits (Agilent). Libraries were pooled and sequenced on an Illumina NextSeq, producing 150 bp, paired-end reads. The Breseq computational pipeline was used to align reads to the reference sequence and identify mutations (Deatherage and Barrick 2014).

### Statistics

R3.5.3 used for plotting and mutation similarity analysis (R Core Team 2017). The mclustcomp function in package mclustcomp was used to estimate Dice’s coefficient of similarity comparing mutations fixed in clones isolated from different evolved populations. Simulations were performed using a custom Python script, which is available on request.

## Supplementary Material

Supplementary data are available at *Molecular Biology and Evolution* online.

## Supplementary Material

msab077_Supplementary_Data
