# Supplementary material for: Historical Contingency Causes Divergence in Adaptive Expression of the lac Operon
Source: Mol Biol Evol. 2021 Mar 21;38(7):2869–79. doi: 10.1093/molbev/msab077 (PMC8233506; doi:10.1093/molbev/msab077)
Supplement: msab077_Supplementary_Data [file msab077_supplementary_data.zip › SI.pdf]

Figure S1. Schematic of the evolution experiment. Six replicate populations were selected in four different environmental treatments lactose only (Lac), a combination of glucose and lactose fluctuating daily (G/L), or a combination of glucose and lactose fluctuating every 2,000 generations with one treatment starting with glucose (G_L) and another with lactose (L_G). Replicate populations were started directly from an ancestor, *Escherichia coli* REL606 except that the L_G treatment was derived from the lactose only treatment at 2,000 generations.

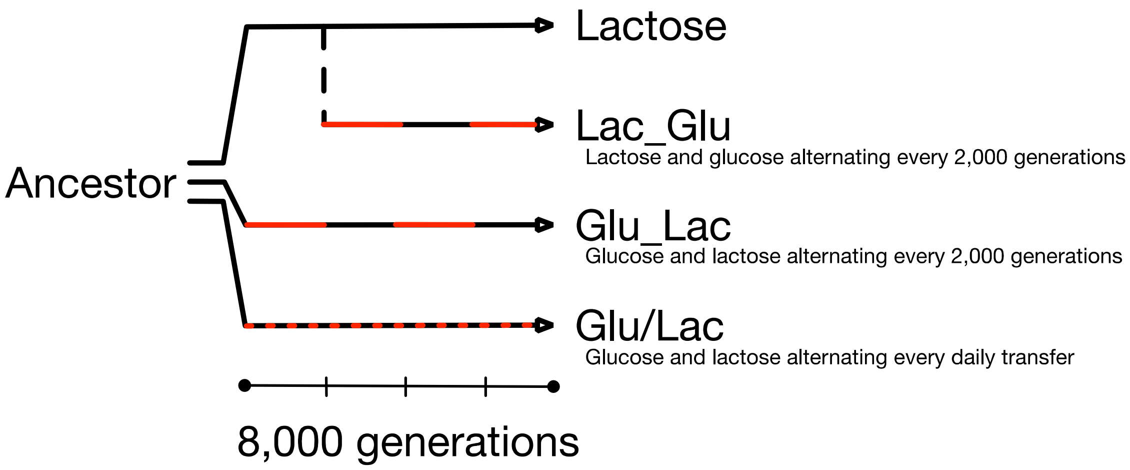

Figure S2. Virtual competitions predict a similar *lacI–* fitness effect to measurements in lactose for Ev^{lacI+} strains. Filled circles show experimental measured fitness effect of mutating *lacI*+ev strains to *lacI–*. Open circles show predicted fitness of *lacI–* vs. *lacI*+ev based on independent growth curves in lactose. Errors are the 95% confidence intervals for both sets of points. Note that due to noise in the growth curve data, model fitting was unstable and some virtual competitions resulted in no growth (or even a decrease in OD) for one strain. We excluded such outliers from the virtual competition predictions.

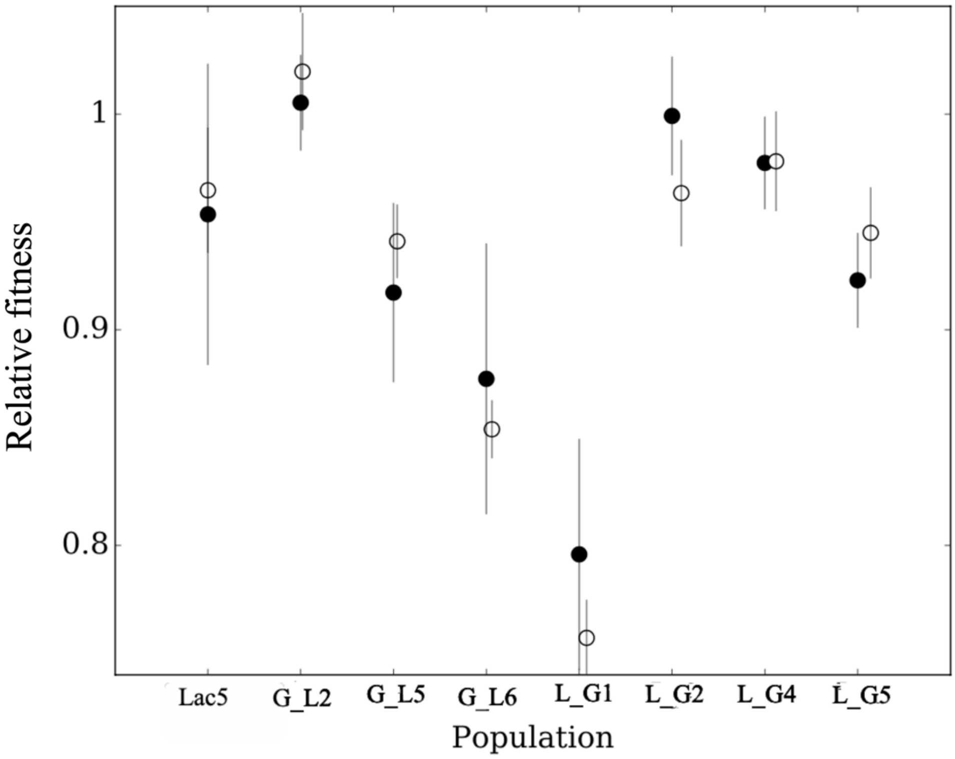

Figure S3. Effect of *lacI*– mutation on growth dynamics of the ancestor and evolved clones. Tested strains are as indicated in each panel. Orange lines indicate that strain with the wild type *lacI* allele encoding a functional repressor and blue lines indicate the same strains except with a *lacI*– mutation casing the repressor to be non-functional. Lines indicate the mean of replicate growth curves (n≥3). All growth curves were performed in the lactose environment.

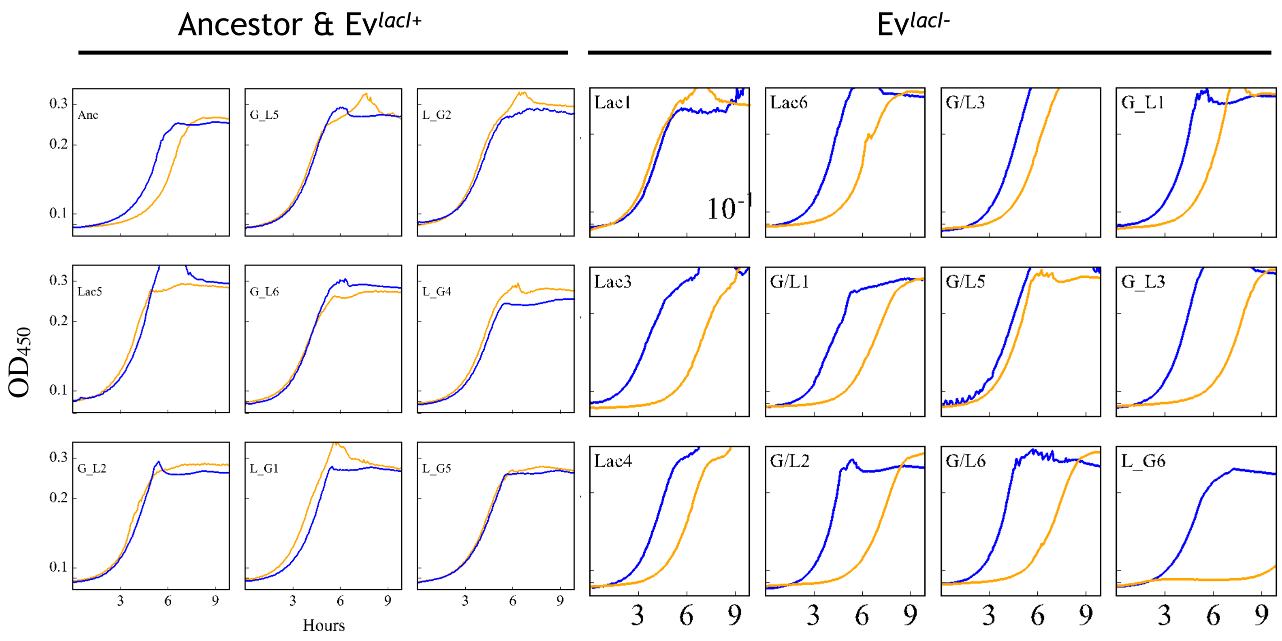

Fig S4. Inducer LacZ enzyme response curves of the ancestor and Ev^{lacI+} strains. Miller assays were carried out to measure expression of the LacZ enzyme measured in Miller Units (MU) at increasing concentrations of the inducer, IPTG. Enzyme activity estimates were carried out when populations were at mid-log ($0.15 < OD_{450} < 0.2$) during growth in DM medium supplemented with 0.2% glycerol. Estimates were normalized by cell density and the rate of cell doubling, to give a measure of promoter activity (Kuhlman et al. 2007). Points represent experimental measurements (black, ancestor; blue, Ev^{lacI+} strains), with standard deviations shown ($n \geq 3$). Hill function model fits are shown as red lines in each panel. For comparison, the ancestor induction-response curve is shown as a grey dashed line in panels of evolved clones.

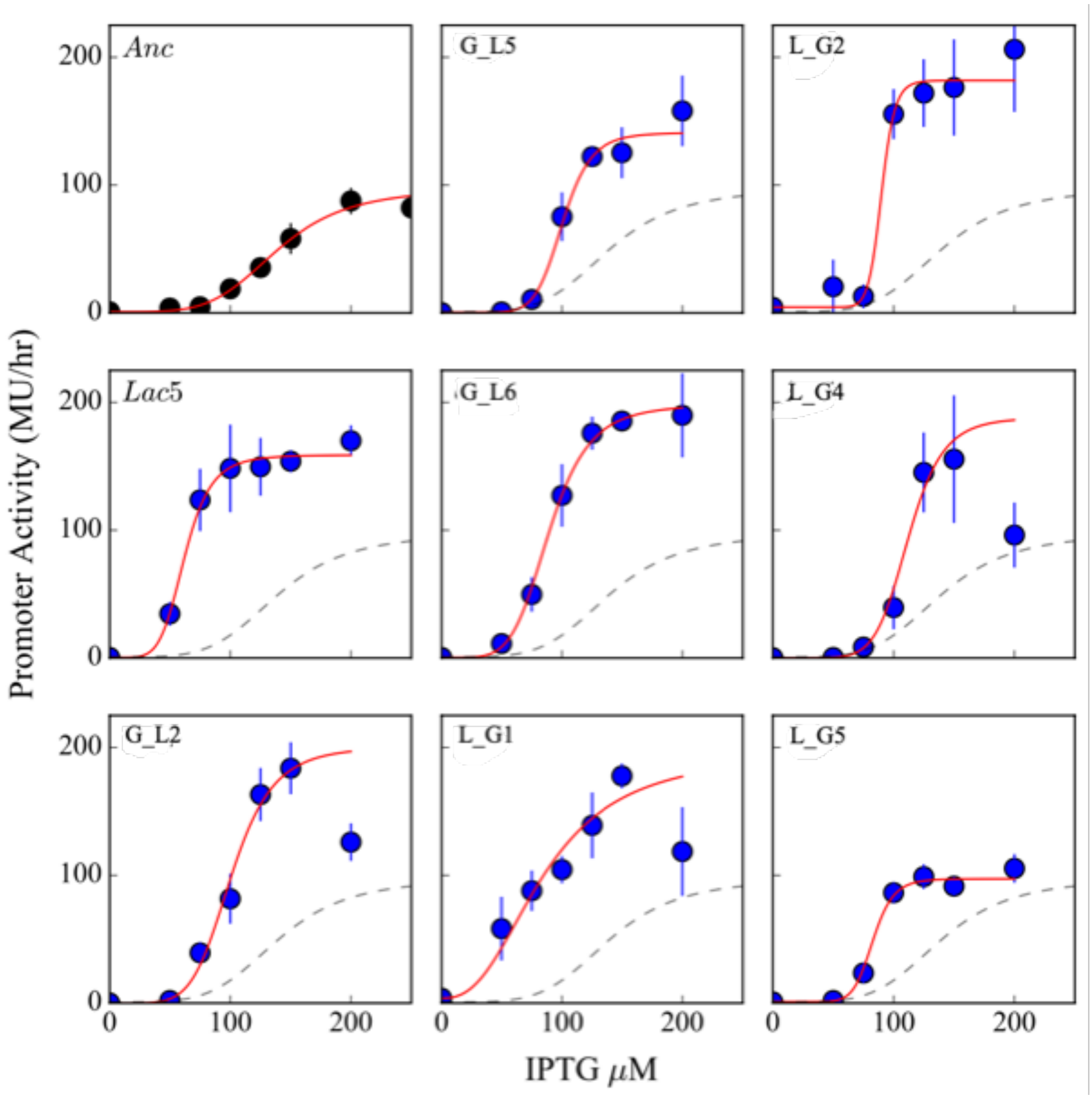

Fig S5. Inducer *lac* promoter response distributions of the ancestor and Ev*lacI+* strains. A P*lac*-GFP reporter was integrated into the *att*Tn*7* site and expression was assessed by flow cytometry at four and six hours following transfer to fresh DM + 0.2% glycerol medium supplemented with noted levels of the inducer, TMG. Note that the ancestor is estimated as having a lag time approximately two hours longer than the evolved strains (Fig 5). Differences in growth rate can be accounted for, therefore, by comparing ancestral expression profiles taken at six hours to the evolved Ev*lacI+*profiles taken at four hours. In this comparison, Ev*lacI+* clones still have a clearly higher P*lac*-*gfp* expression at intermediate inducer concentrations.

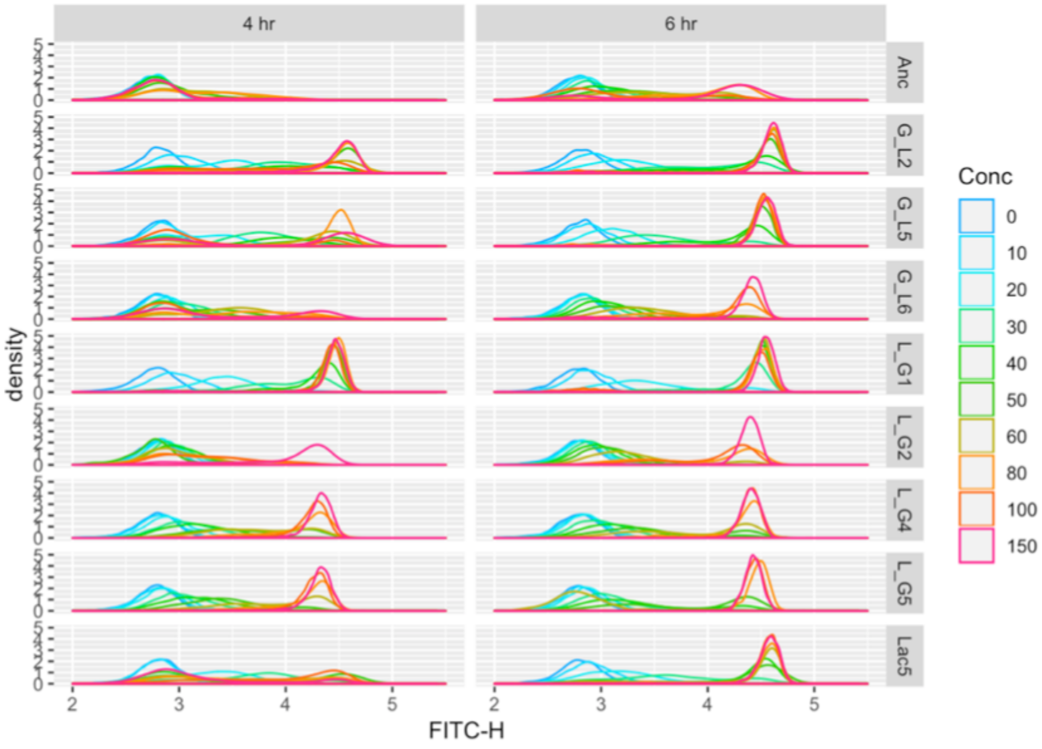

Fig S6. Differential of a *lac* reporter is largely dependent on the LacY permease. A loss-of-function *lacY* mutation was added to ancestor and Ev*lacI+* strains (from the Lac5 population) containing the P*lac*-gfp reporter. Expression of the reporter was measured in DM containing glucose (top row of each panel) or glycerol (bottom row of each panel) and supplemented with concentrations of lactose (A) or TMG (B) inducers. Fluorescence was determined by flow cytometry when populations had reached mid-log growth. We find that the Lac5 evolved strain (TC2845) was more sensitive to TMG than the ancestor (REL606). Expression levels were high at all lactose concentrations used, so that we are unable to determine any difference in induction sensitivity. In the absence of functional LacY (columns labelled '*lacY**', two independently constructed derivatives are reported for each parent strain), neither strain expressed the *lac* reporter at any lactose level. Both strains expressed the reporter at low levels in the presence of TMG and glycerol, but expression was similarly sensitive to TMG concentration.

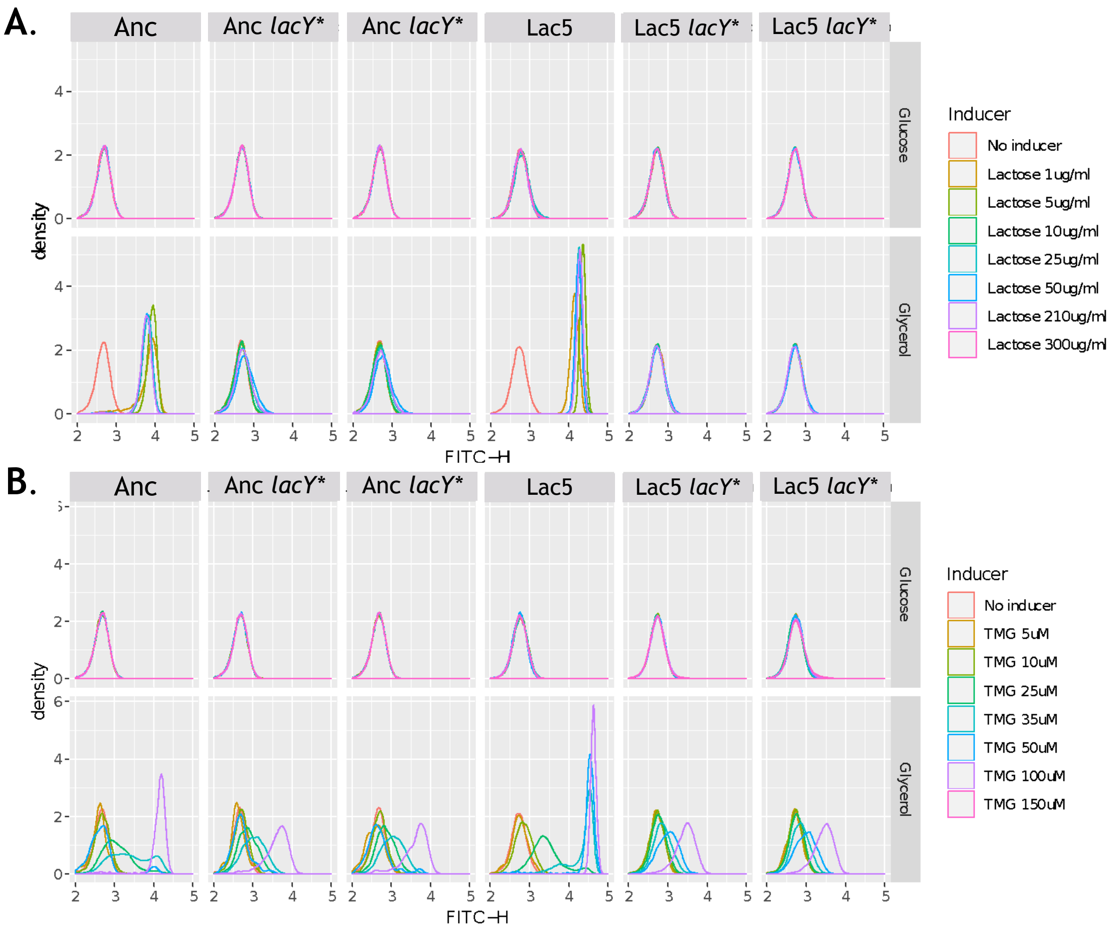

Figure S7. Comparison of genetic parallelism between clones from different population sets. Genetic parallelism was quantified using Dices's coefficient of similarity. This metric assesses parallelism as the number of in common mutated genes over the total number of mutated genes considering a pair of evolved clones. The coefficient was computed for all unique combinations (excluding self-self) of evolved clones and then averaged over combinations within the groups indicated at X-axis tick labels (closed symbols). **A,** comparison of clones based on *lacI* evolved type (i.e., $Ev^{lacI–}$: $Ev^{lacI–}$, $Ev^{lacI–}$: Ev^{lacI+}, Ev^{lacI+}: Ev^{lacI+}). The *lacI* mutation itself was omitted from this analysis. **B,** comparison of clones based on selection environment. Lines show 95% confidence intervals based on 10,000 randomizations where clone label was randomly assigned to each set of mutated genes. Where symbols fall within the confidence intervals indicated by the randomized data there is no indication that mutations in the relevant groups accumulate in different sets of genes. Six mutator populations that had high numbers of presumably non-selected mutations were omitted from these analysis (Lac1, $Ev^{lacI–}$; Lac3, $Ev^{lacI–}$; L_G1, Ev^{lacI+}; L_G3, polymorphic; L_G5 Ev^{lacI+}; L_G6 $Ev^{lacI–}$).

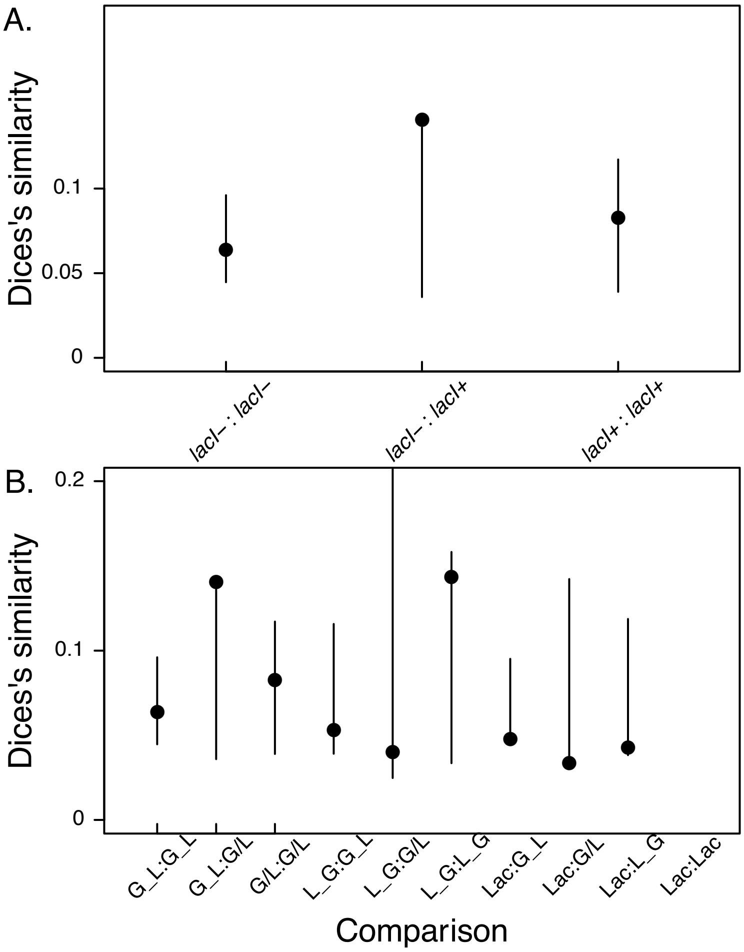

Table S1: Oligonucleotides used in pTarget-*uspB* construction.

ID	Purpose	Sequence (5'-3')
451	*uspB*^{Anc} amplification-f	CTAAGCTTCTGCAGGTCGACCATCTACGTGGATCAGAG
462	*uspB*^{Anc} amplification-r	CGAGTCGGTGCTTTTTTTGAATTCTCTAGAGTTTGTAATCAGTCTCGAGC
452	pTarget backbone amplification-f	GTCGACCTGCAGAAGCTTAG
463	pTarget backbone amplification and incorporation of *uspB* targeting N20-r*	ATTTTAACTTGCTATTTCTAGCTCTAAAACACTGGAAAGCGGGCAGTGAGACTAGTATTATACCTAGGACTGAGC
449	Link pTarget and 461 oligo	ACTTTTTCAAGTTGATAACGGACTAGCCTTATTTTAACTTGCTATTTCTAGCTCTAAAAC
461	Link 461 oligo and *uspB* fragment	AAGGCTAGTCCGTTATCAACTTGAAAAAGTGGCACCGAGTCGGTGCTTTTTTTGAATTCTCTAGA

*Underlined regions of description and oligo sequence region indicate correspondence.

Table S2: The distribution of mutation fitness effects for simulations. Background mutations were segmented into six classes, with fitness effects from −10% to +10%. The Proportion of mutations in each class was chosen to satisfy realistic mean effect (*s*) and proportion of beneficial mutations (*P*ben) as determined in a study comparing observed and simulated evolutionary dynamics of bacterial populations (unpub. obs. K.K., R.B.R.A., T.F.C). Background mutations occur at a genome-wide rate of 7×10^4 $generation^{-1}$ and mutations to *lacI* occur at a rate of 1.72×10^{-7} $cell^{-1}$ $generation^{-1}$ (Fig 3).

**Effect**	**Proportion**
– 10%	0.0475
– 5%	0.235
– 2.5%	0.700
+2.5%	0.01
+5%	0.005
+10%	0.0025
